# A Test Can Have Multiple Reliabilities

**DOI:** 10.1007/s11336-021-09800-2

**Published:** 2021-09-08

**Authors:** Jules L. Ellis

**Affiliations:** Behavioural Science Institute, Radboud University Nijmegen, P.O.B. 9104, 6500 HE, Nijmegen, The Netherlands

**Keywords:** true score, stochastic subject, domain sampling, latent variable, generalizability, reliability, indeterminacy

## Abstract

It is argued that the generalizability theory interpretation of coefficient alpha is important. In this interpretation, alpha is a slightly biased but consistent estimate for the coefficient of generalizability in a subjects x items design where both subjects and items are randomly sampled. This interpretation is based on the “domain sampling” true scores. It is argued that these true scores have a more solid empirical basis than the true scores of Lord and Novick (1968), which are based on “stochastic subjects” (Holland, 1990), while only a single observation is available for each within-subject distribution. Therefore, the generalizability interpretation of coefficient alpha is to be preferred, unless the true scores can be defined by a latent variable model that has undisputed empirical validity for the test and that is sufficiently restrictive to entail a consistent estimate of the reliability—as, for example, McDonald’s omega. If this model implies that the items are essentially tau-equivalent, both the generalizability and the reliability interpretation of alpha can be defensible.

It is an honour that I have this opportunity to comment on the article of Sijtsma and Pfadt (in press). It is interesting to see how the opinion of Sijtsma developed over time. For the superficial reader, the message of the 2009 article seems to be “bad alpha”, while the message of the current article seems to be “hail alpha”. Sijtsma and Pfadt nicely point out that the message of Sijtsma was rather that alpha is a poor index of unidimensionality, but an acceptable lower bound of reliability. Nevertheless, my impression is that Sijtsma and Pfadt current opinion over alpha is more favourable than Sijtsma’s (2009) opinion, especially in comparison with the greatest lower bound.

I agree with the conclusion of Sijtsma (2009) and Sijtsma and Pfadt (in press) that alpha should not be used as an index of unidimensionality. Hoekstra et al. (2019, table 3) show that, in a sample of 534 corresponding authors of nine top-tier journals from four disciplines, 80% made the wrong inference about alpha, suggesting that they interpreted alpha as an index of unidimensionality. I believe the frequent use of the term “internal consistency” is related to this. Using the term “internal consistency” for alpha is misleading, and use of the term in this meaning should be banned from future academic publications. I also agree with the claim of Sijtsma and Pfadt that alpha still has many advantages as an index of reliability, and that the lower bound theorem of Guttman (1945; attribution by Lord and Novick 1968 , p. 87) is valid and useful, which is also the conclusion of Raykov and Marcoulides (2019). However, I do not agree with the conclusion of Sijtsma and Pfadt, that “this is really all there to say about coefficient alpha”. An important perspective on alpha is missing here, namely that it can be interpreted as an estimate of the coefficient of generalizability in a subjects x items design (Cronbach et al. 1972, pp. 80–82; Webb et al. 2006). I will discuss this in the next section. In Sect. 2, I will argue that this interpretation has advantages over the interpretation of alpha as a lower bound of reliability.

## Alpha as an Estimate for the Coefficient of Generalizability

The typical design where alpha is applied is a subjects x items design, where the items are questions or parts of a psychological test. Two different interpretations of alpha are possible here: one based on classical test theory (CTT) (Lord and Novick 1968) and one based on generalizability theory (GT) (Cronbach et al. 1972). Let me briefly explain the difference for readers who are unaware of the difference; see Vispoel et al. (2018) for a more elaborate discussion. In CTT, as described by Lord & Novick (1968, pp. 82-88) in their development of alpha, it is assumed that the test items are, in ANOVA terms, a fixed factor. That is, if the measurement is repeated, it would necessarily be based on exactly the same test items. In GT, in contrast, it is assumed that the items are, in ANOVA terms, a random factor. That is, it is assumed that the items are sampled from a large pool of item, and that if the measurement is repeated, one might use another sample of items, at least in theory. For an example of items that form a random factor, consider an examiner who possesses a pool of 1000 exam items, and who construes an exam each year by drawing 40 items randomly from the pool. For standard psychological tests, it is usually more difficult to replace the items, but still one can imagine that the test constructors could have ended with slightly different items.

In a design of subjects x items, where the items are drawn from a large pool, the item pool would be called the *domain* or *universe* (Nunnally 1978, p. 194), and the* universe score* of a subject would be defined as the expected value of its score across all items in the item pool (Webb et al. 2006). This universe score is also called a true score (Nunnally 1978, p. 194; ), and it will be called the *domain sampling true score* here. The* coefficient of generalizability* is defined as the variance of the universe scores divided by the variance of the test scores (Cronbach et al. 1972, p. 17; Webb et al. 2006). This coefficient of generalizability can be estimated by alpha (Cronbach et al. 1972, pp. 80-82, 98). This then is another advantage of alpha that I would like to add to the discussion of Sijtsma and Pfadt: *coefficient alpha tells you how representative the test scores are for scores that would be obtained with the full domain of admissible items.*

The next question is: how good is alpha as an estimator for the coefficient of generalizability? While Sijtsma discuss the error and bias of alpha when subjects are sampled, in GT alpha will also have an error and bias due to the sampling of items. In the example of the 40 items sampled from a pool of 1000, if alpha is computed each year in the 40-items sample, then the average value of alpha over years would be approximately correct according to Cronbach et al. (1972, p. 98; Cronbach and Shavelson 2004 , p. 402). Thus, alpha is claimed to be an approximately unbiased estimate of coefficient of generalizability even if the items are not essentially $\tau$-equivalent. McDonald (1978), however, showed that alpha is a lower bound to the coefficient of generalizability if the domain has a finite number of common factors, with equality only if the item covariances are equal.

The fact that coefficient alpha can be used as an estimate for the coefficient of generalizability even in the absence of unidimensionality, albeit biased, is certainly an advantage. In some cases, the selection of test items should be based on a broad domain definition rather than a theoretical analysis of dimensionality, and focussing on unidimensionality may lead to tests that are too narrow in content. As Cronbach and Shavelson (2004, p. 413) put it in their comment on the quest for unidimensionality: “A contrary position emphasizes that one needs to represent all aspects of the variable that is the focus of measurement, not narrowing it to a single focal topic”. This is another advantage of alpha that I would like to add to the discussion of Sijtsma and Pfadt: *if the item domain is broad and heterogeneous, a high value of coefficient alpha tells you that you have enough items to cover it.*

Ironically, Sijtsma and Pfadt (in press) defend alpha as an index relevant to reliability by virtue of the lower bound theorem based on CTT true scores, whereas Cronbach (1951, p. 306) adopted the domain sampling interpretation of alpha (“$\alpha$ is therefore an estimate of the correlation expected between two tests drawn at random from a pool of items like the items in this test”) and later disagreed with the lower bound interpretation:

My 1951 article embodied the randomly parallel-test concept of the meaning of true score and the associated meaning of reliability, but only in indefinite language. Once Lord’s (1955) statement was available, one could argue that alpha was almost an unbiased estimate of the desired reliability for this family of instruments. The *almost* in the preceding sentence refers to a small mathematical detail that causes the alpha coefficient to run a trifle lower than the desired value.

This detail is of no consequence and does not support the statement made frequently in textbooks or in articles that alpha is a lower value to the reliability coefficient. (Cronbach and Shavelson 2004, p. 402)

Alpha is a consistent estimator for the coefficient of generalizability in the subjects x items design (Webb et al., p. 16), but it is not directly clear how large the sampling errors can be under the sampling of items, and how this depends on further assumptions such as dimensionality. To illustrate this, I simulated items with a 2-dimensional 2PL model $P(X_{i} = 1|\Theta _{1}, \Theta _{2} ) = (1 + ~\hbox {exp}~(-\alpha _{1i} \Theta _{1} - \alpha _{2i} \Theta _{2} - \beta _{i} )) ^{-1}$ with $\Theta _{1},\Theta _{2}$ bivariate standard normal with correlation 0, and $\alpha _{1i} , \alpha _{2i}\sim Unif(0.5, 2.5) \hbox {and} \beta _{i}\sim Unif(-2, 2),$ where each item loaded on exactly one dimension: $\hbox {min}\{\alpha _{1i} , \alpha _{2i}\} = 0, ~\hbox {max}~\{\alpha _{1i} , \alpha _{2i}\} > 0.$ In 1000 samples, each with a test of 5 randomly selected items and 10,000 subjects, alpha ranged from 0.16 to 0.75. The 5$^{\mathrm {th}}$ percentile of alpha was 0.23, and the 95$^{\mathrm {th}}$ percentile was 0.57. The conclusion is that alpha is not always close to the coefficient of generalizability: for a small number of multidimensional items, the estimation error in alpha may be sizable even if the subject sample is large. Confidence intervals for alpha in the two-way random model are available for normally distributed variables (McGraw and Wong 1996; Demetrashvili et al. 2016), and bootstrap methods are developed for the variance components (Brennan 2007; Tong and Brennan 2007) and their ratio (Gilder et al. 2007; Ye et al. 2020). Still, it would be helpful for test makers in explorative research to have a simple guideline on the a priori minimum test length required for accurate estimation of the coefficient of generalizability with alpha.

## The Indeterminacy of True Scores

Sijtsma and Pfadt (in press) assume in their article the existence of true scores as defined by Lord and Novick (1968). This is common practice in treatments of reliability, and I can see the merits of it, but in the present section, I will argue that these true scores are not well defined in most applications of coefficient alpha, and that this obscures the meaning of the concept “reliability”. The domain sampling true score has a stronger connection to observations, and for this reason, the interpretation of coefficient alpha as an estimate of a coefficient of generalizability should be preferred.

The definition of true scores by Novick (1966) and Lord and Novick (1968, p. 30) can be paraphrased as follows if it is applied to item scores:

### Definition 1

The true score of a subject on an item is the expected value of the observed score, where the observed score is drawn randomly from a probability distribution that depends on the subject and the item.

This definition assumes that there is some probability distribution of scores within a subject, and I will refer to this probability distribution as the *within-subject distribution*, and to the resulting true scores as *stochastic subject true scores.* The term stochastic subject was coined by Holland (1990). Note that this definition does not specify the nature of the randomness of the observed scores. The true score is not necessarily defined by instantaneous replications under the same circumstances, where the subject is “brainwashed” between replications, although Lord and Novick (p. 29) cite this fictitious example of Lazarsfeld. An entirely different example of definition 1, that does not involve stochastic processes inside the subject, is this: if we measure the height of a child on one randomly sampled day from a five-year period, the corresponding true score would not be the momentary height, but rather the average height of the child over the entire period of five year. Indeed, Lord and Novick acknowledge the existence of multiple true scores:

Finally, with respect to the syntactic definition of true score we have adopted here, it should be evident that a person’s true score will depend on the various kinds of conditions under which the measurements are taken. For example, of all the conditions which affect measurement, we might choose to control lighting conditions. Suppose we set up two lighting conditions, one called “good” lighting and the other “bad”. Then, over repeated experimentally independent observations for each condition, a true score for each person will be definable, and presumably these true scores will differ for each person over the two conditions. Also, if a third condition which involves a random sampling of the first two conditions is considered, a third true score can be defined. In Chapter 7 the first two of these true scores will be called a specific true score and the third, a generic true score. (p. 43)

The conclusion is that for the same test item, different true scores exist, depending on the within-subject probability distribution of the circumstances. Lord and Novick (p. 29) require that the within-subject distribution is “well-defined”, and in that case the true scores are well defined. However, the within-subject distribution is often *not* well defined. Lord and Novick (p. 30) write that this distribution “is a hypothetical one because as we noted in Chapter 1, it is not usually possible in psychology to obtain more than a few independent observations”. Even if such independent observations were possible, alpha is routinely applied in situations where only one observation per item per subject is available, and the possibility to do this is often presented as the main advantage of alpha. Thus, in most applications of alpha, the within-subject distribution is ill-defined, and then, the true scores are ill-defined.

That the within-subject distributions are ill-defined is not a logical necessity. In some cases, one can draw a random sample of observations from the same subject, as in the cited example given by Lord and Novick, and in that case the true scores can be properly defined. But in the typical application where coefficient alpha is being used outside GT, such explicit sampling schemes are absent. For example, suppose that a test is administered on one day in one location, and the answers are scored by one rater, and alpha is computed from this. Are the true scores now defined by sampling days within this fixed location and this fixed rater, or by sampling locations within this fixed day and fixed rater, or by sampling raters, or by a combination of days, locations and raters? Each of these possibilities may yield different true scores and different reliabilities, even though they all satisfy Lord and Novick’s definition. The sampling scheme in reliability assessment in CTT is usually not explicit in these facets, leaving the reliability ill-defined. In contrast, GT requires explicit sampling schemes and solves the problem by adopting the domain sampling true score.

This limitation of stochastic subject true scores does not mean that they are useless under all circumstances. Ellis (1993) showed that a factor model for stochastic subject true scores predicts measurement invariance, while this prediction does not follow if the factor model holds merely for latent variable true scores. However, in that case there are additional data and additional restrictions about subpopulations. The point here is that stochastic subject true scores are ill-defined in a subject x items design without additional data or restrictions.

Let me now take this to the extreme by adding a second definition of true scores, in which they are considered as latent variables, possibly in factor analysis or item response theory models:

### Definition 2

For a set of random variables $X_{1},X_{2},...,X_{J}$, true scores are any set of (possibly latent) random variables $T_{1},T_{2},...,T_{J}$ such that, with $E_{i}:=X_{i}-T_{i}$, it holds that $\mathbb {E}(E_{i}|T_{j})=Cov(E_{i},T_{j})=0 ~\hbox {for all}~ i,j=1,...,J.$

I will call this *latent variable true scores*. It should be emphasized here that there is no claim here that these true scores are uniquely defined; on the contrary, many different true score variables may fit this definition with the same observed score variables, similar as in factor score indeterminacy (McDonald 1977; Steiger 1979) and indeterminacy of latent variables in the Rasch model (Ellis and Junker 1997, p. 508). The stochastic subject true scores of Lord and Novick are latent true scores with the additional restriction that $T=\mathbb {E}(X|S)$, where *S* is a variable that identifies the subject. Other latent variable true scores can defined by a random sampling formulation (Holland 1990), meaning that there is no within-subject variability; the additional restriction then is that $X=\mathbb {E}(X|S)$.

We need one more definition:

### Definition 3

For a set of random variables $X_{1},X_{2},...,X_{J}$ with latent variable true scores $T_{1},T_{2},...,T_{J}$, we say that *the errors are uncorrelated* if $Cov(E_{i},E_{j})=0$ for all $i,j=1,...J \,\text{ with }\, i \ne j.$

Latent variable true scores with uncorrelated errors will always exist, however. Suppes and Zanotti (1981)showed that if $$X_{1},X_{2},...X_{J} $$ are binary random variables with a joint distribution, there exists a random variable $$\Theta $$ such that $$X_{1},X_{2},..., X_{J}$$ are conditionally independent given $$\Theta $$. They claim that this can easily be extended to continuous variables. For such $$\Theta $$, we can define $$T_{i}:=\mathbb {E}(X_{i}|\Theta )$$ and $$E_{i}:=X_{i}-T_{i}$$, to obtain $$\mathbb {E}(X_{i}-T_{i}|T_{j})=\mathbb {E}(\mathbb {E}(X_{i} -T_{i}|T_{j},\Theta )|T_{j})=\mathbb {E}(0|T_{j})=0$$, and uncorrelated errors follow in a similar fashion from conditional independence given $$\Theta $$. In other words, the assumption that latent variable true scores exist with uncorrelated errors is always true.

For given latent variable true scores with uncorrelated errors, the lower bound theorem, attributed to Guttman (1945) by Lord and Novick (1968, p. 87), says that alpha is less than or equal to the reliability. The proof has been given in many texts and will not be repeated here. The following proposition illuminates that the latent variable true scores are so ambiguous that we can always assume that they are such that the reliability is greater than or equal to alpha; the restriction of uncorrelated errors is trivially satisfied if there are no further restrictions on the true scores. Let $X_{+}:=\Sigma _{i}X_{i}$ and $T_{+}:=\Sigma _{i}T_{i}$ and $Rel(X_{+}):=Var(T_{+})/Var(X_{+})$ .

### Proposition 1

If a set of random variables $X_{1},X_{2},...,X_{J}$ has covariance matrix *C* and $Var(X_{+})>0$, then there exist latent variable true scores $T_{1},T_{2},...,T_{J}$ such that the errors are uncorrelated and such that $\alpha \le Rel(X_{+})$ holds for these true scores.

### Proof

Define $T_{i}:=X_{i}$, then $E_{i}=0$ and therefore $Cov(E_{i},E_{j})=0$ for all $i,j=1,...,J$. Because $E_{i}=0$, we have $Rel(X_{+})=1$, and it follows that $\alpha \le Rel (X_{+})$ . The existence *C* of is merely required in order to assure that all relevant moments exist. $\square$

Note that it can even be assumed that the reliability is equal to 1 without contradicting the CTT restrictions; there is no way to disprove this assumption. The additional restriction that the true scores be stochastic subject true scores, i.e. $T=\mathbb {E}(X|S)$ , does not change this if the within-subject distribution is left unspecified or not estimable. Thus, in practical applications outside GT, after only a single test administration, without further model restrictions, one can always assume true scores such that alpha is a lower bound to the thus defined reliability.

## Discussion

Coefficient alpha is typically used in a single test administration with a subjects x items design, and it was argued that in these designs the definition of true scores is ambiguous if the “syntactic” definition of Lord and Novick (1968, p. 30) is being used. Various CTT true score concepts have been discussed, and they all have limitations in this design (these points are certainly not new, and they are paraphrases of arguments brought up by psychometricians in personal communication, including the late Roderick P. McDonald):

- The stochastic subject true scores, which are used by Lord and Novick (1968) are ill-defined. They are defined by a within-subject distribution of scores on one item, while we typically have only one observation from this distribution. Consequently, they are indistinguishable from latent variable true scores in this design.
- The latent variable true scores suffer from indeterminacy. For items with finite range, one can always assume the existence of true scores as latent variables $T_{j}$ with the restrictions $\mathbb {E}(E_{i}|T_{j})=0$ and $Cov(E_{i},T_{j})=0$ and $Cov(E_{i}, E_{j})=0$. These true scores become meaningful only if further restrictions are added, as in factor analysis, or if further data are added, as in GT.

Consequently, a test can have multiple reliabilities, depending on the universe of generalization—which remains implicit in CTT—and the model restrictions—such as the number of common factors. In contrast, the domain sampling true score, called the universe score in GT, is based on a distribution from which multiple observations are available, namely the various item scores of the subject. The domain sampling true scores thus have a much stronger empirical anchor than the other true scores. This true score also has a disadvantage:

- The domain sampling true scores assume that items are randomly sampled from a large domain, which is usually “not true in any strict sense” (Cronbach and Shavelson 2004, p. 404)

Cronbach and Shavelson dismiss this criticism on the same grounds as used by Sijtsma and Pfadt (in press) to dismiss criticism on the use of alpha despite violations of essential -equivalence, namely that models “never fit the data perfectly”.

Everything considered, my evaluation is that the best true scores are either 1) latent variable true scores in the context of a latent variable model with undisputed empirical validity for the given test and with sufficient restrictions to allow consistent estimation of the reliability or 2) domain sampling true scores. In the context of an undisputedly valid latent variable model, one should presumably prefer the measure entailed by the model, such as McDonald’s omega. If the undisputed model implies essential -equivalence, this measure can be coefficient alpha, and in that case both the GT and the CTT interpretation of alpha are defensible. Otherwise, if the model implies uncorrelated errors, then the lower bound theorem would apply, but why would anyone use alpha if the undisputed model entails a consistent estimate that is different from alpha? Such a model can, in theory, have correlated errors, and then coefficient alpha can be greater than the reliability. Coefficient alpha would be useful in situations without such undisputed models, and in these situations, the domain sampling true scores would be best. *For this reason, I advocate the interpretation of coefficient alpha as an estimate for the coefficient of generalizability in a subjects x items design, and I recommend to take this as the default interpretation in teaching and empirical research where the coefficient is reported.*

The discussion of Sijtsma and Pfadt (in press) about the Bentler (2009) model can be analysed by noting that both the common factor and the sum of the common factor and the specific factor are latent variable true scores. Therefore, I agree with Sijtsma and Pfadt’s conclusion that reliability based on the common factor model is a CTT reliability. It is a nice illustration of the fact that a test can have two different reliabilities that both fit within the CTT definition of reliability.
